# The Role of the Medial Prefrontal Cortex in the Conditioning and Extinction of Fear

**DOI:** 10.3389/fnbeh.2015.00298

**Published:** 2015-11-09

**Authors:** Thomas F. Giustino, Stephen Maren

**Affiliations:** Department of Psychology and Institute for Neuroscience, Texas A&M UniversityCollege Station, TX, USA

**Keywords:** prelimbic, infralimbic, freezing, fear, extinction

## Abstract

Once acquired, a fearful memory can persist for a lifetime. Although learned fear can be extinguished, extinction memories are fragile. The resilience of fear memories to extinction may contribute to the maintenance of disorders of fear and anxiety, including post-traumatic stress disorder (PTSD). As such, considerable effort has been placed on understanding the neural circuitry underlying the acquisition, expression, and extinction of emotional memories in rodent models as well as in humans. A triad of brain regions, including the prefrontal cortex, hippocampus, and amygdala, form an essential brain circuit involved in fear conditioning and extinction. Within this circuit, the prefrontal cortex is thought to exert top-down control over subcortical structures to regulate appropriate behavioral responses. Importantly, a division of labor has been proposed in which the prelimbic (PL) and infralimbic (IL) subdivisions of the medial prefrontal cortex (mPFC) regulate the expression and suppression of fear in rodents, respectively. Here, we critically review the anatomical and physiological evidence that has led to this proposed dichotomy of function within mPFC. We propose that under some conditions, the PL and IL act in concert, exhibiting similar patterns of neural activity in response to aversive conditioned stimuli and during the expression or inhibition of conditioned fear. This may stem from common synaptic inputs, parallel downstream outputs, or cortico-cortical interactions. Despite this functional covariation, these mPFC subdivisions may still be coding for largely opposing behavioral outcomes, with PL biased towards fear expression and IL towards suppression.

## Introduction

Pavlovian fear conditioning is a form of learning that serves as a robust model to explore the neurobiological underpinnings of disorders of fear and anxiety, including post-traumatic stress disorder (PTSD). In a typical rodent experiment, an innocuous conditioned stimulus (CS; e.g., an auditory tone) is paired with an aversive unconditioned stimulus (US; e.g., a mild electric footshock). After one or more conditioning trials, presentation of the CS alone comes to elicit a conditioned fear response (CR) that includes freezing behavior (i.e., immobility except that necessary for respiration), changes in heart rate and respiration, and potentiated acoustic startle (Davis, 1992; LeDoux, 2000; Maren, 2001). Importantly, these fear CRs can be extinguished by repeated presentations of the CS in the absence of the US. In rodents and humans alike, CRs to an extinguished CS tend to return under a number of conditions including the passage of time (spontaneous recovery), when the CS is presented outside the extinction context (renewal), or with exposure to an unsignaled US (reinstatement; Bouton, 2000, 2002; Hermans et al., 2006; Maren et al., 2013; Vervliet et al., 2013; Goode and Maren, 2014). These recovery or relapse phenomena suggest that extinction does not erase fear memories, but generates a new safety memory that inhibits the expression of fear. In addition, extinction learning itself is a fragile process, dependent on many factors including timing relative to conditioning (Maren and Chang, 2006; Myers et al., 2006; Maren, 2014) and stress (Maren and Holmes, 2015).

While learned fear serves an adaptive purpose aiding survival, pathological fear states are thought to underlie various stress and trauma-related disorders such as PTSD, which has a lifetime prevalence of nearly 8% in the general population (Kessler et al., 1995, 2005). Not surprisingly, this number increases to as high as 30% in combat-exposed veterans (Koenen et al., 2008), amplifying the need for more effective therapies. PTSD has been described as the only mental health disorder with a known cause (i.e., a traumatic experience; Pitman et al., 2012) and is characterized by heightened arousal and resistance to extinction learning (Rauch et al., 2006). Many have argued that PTSD may, at least in part, be a disorder of the fear circuitry (Shin and Handwerger, 2009) and an enhanced understanding of learned fear is relevant to the psychological processes underlying this disorder (Liberzon and Sripada, 2008; VanElzakker et al., 2014). It is possible that PTSD patients exhibit exaggerated fear conditioning, resistance to extinction, or both; ultimately, they exhibit persistent fear CRs (Pitman, 1988).

Due to the prevalence and debilitating nature of stress and trauma-related disorders, there has been a surge in interest in understanding the neural processes subserving learned fear and its subsequent extinction (Quirk and Mueller, 2008; Milad and Quirk, 2012; Maren et al., 2013). A triad of brain regions, including the amygdala, hippocampus and medial prefrontal cortex (mPFC) has been heavily studied in relation to fear (Maren and Quirk, 2004; Herry et al., 2010; Dejean et al., 2015). While it is well accepted that the amygdala and hippocampus play a role in conditioned fear and extinction, a dichotomy of function has been proposed within the mPFC in which the prelimbic (PL) and infralimbic (IL) cortices regulate the expression and suppression of fear, respectively (Quirk and Mueller, 2008; Sotres-Bayon and Quirk, 2010; Milad and Quirk, 2012; Maren et al., 2013). Here, we critically review the anatomical and physiological evidence that has led to this proposed dichotomy of function within mPFC, comparing results from rodents with those in humans.

## The Fear Circuit

It is well established that both the acquisition and extinction of fear memories requires synaptic plasticity within the amygdala, however a comprehensive discussion of the amygdala circuitry is beyond the scope of this review (Fanselow and LeDoux, 1999; LeDoux, 2003; Maren and Quirk, 2004; Herry et al., 2010; Pape and Pare, 2010; Lee et al., 2013; Duvarci and Pare, 2014). The amygdala is a node of highly interconnected nuclei; the basolateral complex of the amygdala (BLA; consisting of the lateral, basal and basomedial nuclei) and the central nucleus of the amygdala (CeA; consisting of lateral and medial components) play critical roles in the acquisition of both fear and extinction memories. It has been suggested that inhibitory neurons within the amygdala play a role in regulating fear output. These include: (1) the intercalated cell masses (ITCs) positioned between the BLA and CeA (Nitecka and Ben-Ari, 1987; McDonald and Augustine, 1993; Paré and Smith, 1993; Royer et al., 1999; Lee et al., 2013; Duvarci and Pare, 2014); (2) local inhibitory interneurons within the BLA (Spampanato et al., 2011; Wolff et al., 2014); and (3) inhibitory interneurons in CeL that project to CeM (Ciocchi et al., 2010; Haubensak et al., 2010).

How one structure supports the formation and storage of opposing memories is not fully understood, although it appears that distinct cell populations within the BLA may preferentially encode low and high fear states (Goosens et al., 2003; Hobin et al., 2003; Herry et al., 2008; Senn et al., 2014). For example, lesions of the lateral amygdala (LA), a locus for CS and US convergence, or the CeA disrupt fear conditioning (LeDoux et al., 1990; Goosens and Maren, 2001; Wilensky et al., 2006). Similarly, reversible inactivation of the BLA prevents the acquisition and expression of conditioned fear (Helmstetter and Bellgowan, 1994; Muller et al., 1997), suggesting a large degree of overlap between the subnuclei of the amygdala. Studies using overtraining procedures have demonstrated that amygdala lesions disrupt fear memories, not the ability of animals to emit conditioned fear responses (Maren, 1998, 1999). Single-unit recordings have demonstrated learning-related changes in short-latency (less than 15 ms) CS-evoked responses in the LA after fear conditioning, suggesting that these changes are mediated by direct thalamo-amygdala projections (Quirk et al., 1995; Maren, 2000). Moreover, these conditioning-induced changes in spike firing are specifically related to the associative nature of the CS, indicating that the LA is a crucial site of plasticity for fear memories independent of freezing behavior (Goosens et al., 2003). In contrast, the CeA is primarily thought of as an output station, relaying information to the brain stem, hypothalamus and periaqueductal gray (PAG) to initiate fear responses such as freezing (Paré et al., 2004). Whereas the CeL is necessary for fear acquisition, CRs are mediated by CeM output (Ciocchi et al., 2010; Haubensak et al., 2010). Curiously, while the LA encodes CS-US information, there are no direct connections between the LA and CeA to directly mediate fear output, suggesting that the BL or BM or both may act as an interface (Amano et al., 2011). Interestingly, post-conditioning lesions of the basal nuclei block fear expression while leaving learning intact (Anglada-Figueroa and Quirk, 2005; Amano et al., 2011). Selective inactivation of either BM or BL alone was not sufficient to mimic this effect, whereas inactivation of both BM and BL was sufficient. This implies that some level of functional overlap exists between these two regions (Amano et al., 2011).

Additionally, several studies have shown that BLA synaptic plasticity is crucial for the acquisition of extinction (Falls et al., 1992; Lu et al., 2001; Herry et al., 2006, 2008; Kim et al., 2007; Sotres-Bayon et al., 2007). Upon extinction learning, LA neurons typically show a reduction in CS-evoked neural activity (Quirk et al., 1995; Repa et al., 2001). However, a distinct population of LA cells maintain CS-evoked responding throughout extinction learning (Repa et al., 2001). Interestingly, after extinction, patterns of CS-evoked neural activity in LA are mediated by the context and reflect the level of freezing (i.e., larger responses occur when fear renews; Hobin et al., 2003). In summary, there is compelling evidence to support the notion that the amygdala is a crucial locus for the acquisition and extinction of learned fear with both “fear” and “extinction” neurons existing within the same subnuclei whose CS-evoked activity strongly correlates with the level of fear expression (Quirk et al., 1995; Repa et al., 2001; Goosens et al., 2003; Herry et al., 2008; Senn et al., 2014).

The hippocampus has also been identified as a key mediator of learned fear. Given the role of the hippocampus in encoding contextual and spatial information it is not surprising this region plays a substantial role in the fear circuit. Numerous studies have shown that hippocampal lesions dampen fear to a context previously associated with a shock US (Selden et al., 1991; Kim and Fanselow, 1992; Phillips and Ledoux, 1992). Importantly, hippocampal lesions produce larger deficits when made soon after context conditioning, suggesting that recent memories rely more heavily on the integrity of the hippocampus (Maren et al., 1997; Anagnostaras et al., 1999). Interestingly, hippocampal lesions do not necessarily interfere with context conditioning when damage is made prior to training (Maren et al., 1997; Frankland et al., 1998), although deficits in the acquisition of contextual fear can be obtained with single-trial procedures (Wiltgen et al., 2006). Collectively, these results suggest that the hippocampus is required for forming and storing memories of the context, but not necessarily context-US associations (Young et al., 1994). These findings support the notion that the hippocampus plays a key role in both the acquisition and expression of conditioned fear to a particular context.

As mentioned above, the extinction of fear is highly context-dependent; that is, fear returns or “renews” when the CS is presented outside the extinction context. Considerable evidence indicates that the renewal of fear is mediated by the hippocampus (Bouton, 2000, 2002; Bouton et al., 2006; Hermans et al., 2006; Maren et al., 2013; Vervliet et al., 2013; Goode and Maren, 2014). For example, many studies have shown that hippocampal inactivation dampens fear renewal when the CS is presented outside of the extinction context (Holt and Maren, 1999; Corcoran and Maren, 2001; Hobin et al., 2006; Maren and Hobin, 2007; Zelikowsky et al., 2012). In addition, disconnections of the hippocampus from the amygdala or prefrontal cortex impair renewal (Orsini et al., 2011), amygdala neurons engaged during fear renewal receive hippocampal and prelimbic input (Knapska et al., 2012) and individual hippocampal neurons expressing Fos after fear renewal preferentially project to both the amygdala and prefrontal cortex (Jin and Maren, 2015). These data suggest that the hippocampus integrates contextual information during conditioning and likely regulates the context dependent recall of fear after extinction learning.

Fear regulation must be tightly controlled and this is thought to depend on the mPFC. Two subdivisions of mPFC in rodents, and their human homologs, have been identified as having distinct roles within the fear circuit. The prelimbic cortex (PL) is thought to regulate fear expression, whereas the infralimbic cortex (IL) mediates fear suppression (Quirk and Beer, 2006; Sotres-Bayon and Quirk, 2010; Milad and Quirk, 2012; Riga et al., 2014). A similar division of labor has been proposed in humans, indicating that the neural mechanisms of extinction learning may be conserved across species (Phelps et al., 2004; Schiller et al., 2008; Sehlmeyer et al., 2009; Milad and Quirk, 2012; Vervliet et al., 2013). Below we review the extant literature that has led to this proposed dichotomy of function.

## Anatomy of the Rodent mPFC

Initially, the PFC was defined by a granular layer IV; this criterion excluded lower level mammalian species, including rodents (Brodmann, 1909). This classification was challenged by Rose and Woolsey, who suggested that projections from the mediodorsal nucleus of the thalamus were the defining feature of the PFC. This re-definition of the PFC was inclusive of all mammalian species (Rose and Woolsey, 1948) and it is now generally accepted that rodents have a PFC with some homology to that of higher-order species (Uylings and van Eden, 1990; Uylings et al., 2003). These homologies are based on several criteria including cytoarchitectonics, connectivity patterns, electrophysiological properties, protein expression, and changes in behavior following damage (Campbell and Hodos, 1970; Uylings and van Eden, 1990; Uylings et al., 2003). Indeed, the rodent PFC like that in humans plays a role in an array of complex behaviors (Heidbreder and Groenewegen, 2003; Kesner and Churchwell, 2011).

### Laminar Organization and Cell Types

In rodents, the mPFC is identified as the agranular portion of the frontal lobe and is divided into three subdivisions: the anterior cingulate (ACC), the PL and the IL. Here, we will primarily focus on PL and IL. The rodent PFC exhibits laminar organization with deep and superficial layers (Caviness, 1975; Yang et al., 1996; Uylings et al., 2003; van de Werd et al., 2010), although a granular layer IV is less well defined when compared to humans and non-human primates (Krettek and Price, 1977b; Uylings and van Eden, 1990; Uylings et al., 2003). PL and IL are neighboring structures, with PL lying just dorsal to IL, which can be distinguished based on cytoarchitectonic features and laminar organization. For example, layer V of PL is less well organized compared to more dorsal regions (i.e., ACC), whereas layer VI cells are arranged in a horizontal fashion in both rats and mice (van de Werd et al., 2010). Due to the relatively large size of PL, layer II and III appear broad compared to neighboring subdivisions. Interestingly, there is evidence for a changing organization along the dorsal-ventral axis of PL, which may transition into IL (Heidbreder and Groenewegen, 2003; Perez-Cruz et al., 2007; van de Werd et al., 2010). This distinction is mainly based on the expansion of layer II at the expense of layers III and V along this axis. In contrast to PL, IL layer II neurons innervate layer I at a much higher rate, making IL layer II appear broad (Krettek and Price, 1977b; van de Werd et al., 2010). While the more superficial layers II and III are easily discernible from a lighter layer V in PL, IL layers are less distinct (Krettek and Price, 1977b). In general, IL layers II-VI have a relatively homogenous layout in terms of cell size and density, with smaller cell bodies compared to PL (van Eden and Uylings, 1985; van de Werd et al., 2010). The contribution of different layers and functional changes along the dorsal-ventral axis of PL and IL are largely unknown, but may be differentially engaged in the fear circuit, similar to the findings noted above regarding distinct populations within amygdala nuclei regulating opposing fear states.

Cortical processing of information requires complex interactions between a number of distinct cell types that fall into two broad categories: principal cells (80–90%) and interneurons (10–20%; DeFelipe and Fariñas, 1992; Gabbott et al., 2005). Neurons are typically classified based on unique characteristics including cell size and shape, dendritic arborization, molecular markers, and connectivity. Pyramidal cells are typically thought to communicate to long-distance targets and are found in layers II-VI (DeFelipe and Fariñas, 1992), although there are noted differences in the firing properties, cell body size and dendritic morphology within and across layers (Yang et al., 1996; Barthó et al., 2004; Molnár and Cheung, 2006; Wang et al., 2006; Otsuka and Kawaguchi, 2008; Brown and Hestrin, 2009; Dembrow et al., 2010; van Aerde and Feldmeyer, 2015). In addition, a number of molecular markers have been identified to categorize specific subclasses of pyramidal cells (Gong et al., 2003; Hevner et al., 2003; Gray et al., 2004; Molnár and Cheung, 2006; Watakabe et al., 2007). The complexity and organization of cortical pyramidal neurons makes the PFC well suited to regulate several functions and an array of behaviors (Heidbreder and Groenewegen, 2003; Kesner and Churchwell, 2011).

Similar to pyramidal cells, interneurons of the cortex are separated into several classes based on unique physiological, morphological, and immunocytochemical markers (Kawaguchi and Kubota, 1993, 1997; Kawaguchi, 1995; Gupta et al., 2000; Ascoli et al., 2008; Povysheva et al., 2008). While sparse in number relative to pyramidal cells, interneurons nonetheless serve to modulate cortical function. Broad classes of interneurons, based on the heterogeneous expression of calcium-binding proteins and neuropeptides such as parvalbumin (PV), somatostatin, vasoactive intestinal polypeptide and cholecystokinin, have been observed in most layers of rodent PFC, although this distribution may not be uniform (DeFelipe, 1993; Kawaguchi and Kubota, 1993, 1997; Kawaguchi, 1995; Gabbott et al., 1997). These distinct classes of interneurons exhibit unique firing patterns, synapsing on specific morphological subregions of pyramidal cells. For example, somatostatin-positive interneurons typically innervate pyramidal cell dendrites to modulate the gain of inputs terminating within those subregions (Kawaguchi and Kubota, 1997; Gupta et al., 2000; Freund and Katona, 2007). In contrast, fast-spiking parvalbumin-positive interneurons (PVINs) target the perisomatic region of pyramidal cells, thereby influencing firing rate and action potential synchronization (Cobb et al., 1995). Interestingly, PV expression in mPFC is generally similar between PL and IL, suggesting the mechanisms for modulating mPFC output are similar between these two brain regions (Gabbott et al., 1997; van de Werd et al., 2010). As with the vast array of principal neurons, the differential contribution of specific subtypes of interneurons within and between mPFC layers within the fear circuit are questions of high interest that remain to be resolved.

#### Inputs

It is well established that PL and IL receive excitatory inputs from regions including, but not limited to, the midline thalamus, BLA, hippocampus and contralateral mPFC (Krettek and Price, 1977a; Little and Carter, 2012, 2013). The posterior portion of the amygdala strongly projects to both PL and IL with sparse innervation from the anterior regions (Krettek and Price, 1977a). Some studies however, have shown strong connectivity from anterior regions, especially from the BLA (Sarter and Markowitsch, 1984; McDonald, 1987). BLA projections synapse on layers II–VI with a small percentage of these projections targeting PVINs (Gabbott et al., 2006). Thus, BLA projections can functionally modulate mPFC output via feed-forward inhibitory mechanisms. In addition, dorsal and ventral hippocampus (CA1/subiculum) exhibit robust excitatory projections to PL and IL (Swanson, 1981; Jay et al., 1989; Jay and Witter, 1991; Azuma and Chiba, 1996; Hoover and Vertes, 2007). These projections have been reported to terminate in all layers of mPFC, although this may shift in density along the dorsal-ventral axis (Jay et al., 1989; Jay and Witter, 1991). In addition, a population of ventral CA1 neurons innervates IL layers I and V and these same hippocampal neurons also synapse on entorhinal neurons, which may be important for integrating contextual and spatial information (Swanson, 1981). Similar to the amygdala, some hippocampal projections may preferentially target mPFC interneurons, inhibiting mPFC output to downstream targets (Sotres-Bayon et al., 2012). In summary, PL and IL receive many similar input patterns, suggesting that these two subdivisions of mPFC integrate incoming information from multiple sources to drive appropriate behavioral responding.

#### Outputs

The regulation of fear is thought to rely heavily on the integrity of the mPFC, which functions to exert top down control over subcortical structures, coding for appropriate behavioral responses. The most widely accepted view is that PL and IL project broadly to the same region (e.g., the amygdala) but to distinct populations of cells that ultimately dictate CRs. To this end, PL and IL both strongly innervate the BLA and these glutamatergic projections originate from layers II, V and VI (DeFelipe and Fariñas, 1992; Pinto and Sesack, 2000, 2008; Gabbott et al., 2005; Hoover and Vertes, 2007). In terms of their potential functional opposition, PL projections terminate in the BLA whereas IL projects to the ventral region of the LA, the basomedial nucleus, and the lateral central nucleus (McDonald et al., 1996; McDonald, 1998; Vertes, 2004). Although many have proposed that IL projections to the ITCs gate CeA output (Royer et al., 1999; Royer and Paré, 2002; Likhtik et al., 2005), recent data challenge this possibility (Cassell and Wright, 1986; Gutman et al., 2012; Pinard et al., 2012; Strobel et al., 2015). Pinard et al. (2012) have suggested that if this indeed is the pathway mediating fear inhibition, it must work via sparse connections. These weak connections may partially explain why extinction learning is not always robust and prone to relapse. Similar results using diffusion tensor imaging and structural tract-tracing techniques in mice further demonstrate largely indistinguishable amygdalar projections from PL and IL (Gutman et al., 2012), although little is known about the functional aspects of PL innervation of the ITCs. One possibility is that IL mediated excitation of the ITCs is disynaptic, acting through the BLA (Strobel et al., 2015). In addition, PL and IL have direct projections the PAG (Hardy and Leichnetz, 1981; Beitz, 1982; Sesack et al., 1989; Floyd et al., 2000; Vianna and Brandão, 2003; Hoover and Vertes, 2007). Floyd et al. (2000) have suggested that rostral PL/IL preferentially innervate the ventrolateral PAG, whereas more caudal portions of PL/IL innervate the dorsolateral PAG. It remains possible that mPFC projections can bypass the amygdala to directly influence freezing behavior. In summary, recent anatomical evidence suggests that PL and IL display overlapping connections, especially to the amygdala and very weakly innervate the ITCs. The majority of these findings are from behaviorally naïve animals however. It would be advantageous to explore the functional outcome of these overlapping projections throughout stages of aversive learning.

#### mPFC Intrinsic Connectivity

A key question in mPFC function revolves around cortico-cortical interactions, which originate from superficial layers II and III (Hoover and Vertes, 2007). While this has not been studied extensively in fear, in slice preparations IL has higher frequency local field potential (LFP) components than PL, and these differ when the two regions are disconnected—implying some level of functional connectivity regulating basal activity (van Aerde et al., 2008). In addition, optogenetic activation of IL inhibits PL pyramidal cells *in vivo* (Ji and Neugebauer, 2012). This feed-forward inhibition may be a necessary component of extinction learning, although this has not been tested. Difficulty arises when addressing these questions simply due to the physical proximity of PL and IL, and the trouble of restricting infusions solely to one region.

## Early Evidence for a Division of Labor

### Lesion Studies

One of the first studies to examine the role of mPFC in defensive behaviors showed that damage to this structure had no effect on flight, biting or reactivity to handling in wild rats, although these lesions primarily encompassed more dorsal regions than PL and IL (i.e., ACC; Divac et al., 1984). In contrast to this report, dmPFC lesions (encompassing ACC/dorsal PL) in laboratory rats increased reactivity to an aversive stimulus and it was shown that these animals were capable of maintaining long-term fear, suggesting that dmPFC is not necessary for memory formation and retention or fear expression (Holson, 1986). More recent work, however, has shown that pre-training ACC lesions impair fear acquisition, while leaving fear expression intact in laboratory rats, although this deficit could be overcome with additional training (Bissière et al., 2008). In a separate study, Morgan et al. (1993) demonstrated that pre-conditioning mPFC lesions (encompassing ACC, PL, and IL) did not have an appreciable effect on the rate of acquisition or level of fear expression to either context or cued fear conditioning. However, these animals took longer to reach extinction criterion, suggesting that mPFC neural activity plays a role in extinction learning (Morgan et al., 1993). In a follow up study, selective PL lesions (damage was mainly restricted to dorsal PL) produced a general increase in both cued and context fear during acquisition and extinction phases, suggesting that dmPFC lesions yield a general *increase* in fear (Morgan and LeDoux, 1995). The authors suggest that these findings revealed a differential contribution of PL vs. IL to the expression of conditioned fear. However, based on the extent of the lesions presented in each study, an alternative interpretation is that behavioral differences reflected gross differences in functions mediated by the dorsal-ventral axis of mPFC and not specifically PL vs. IL. In support of this, some studies have reported *decreased* freezing and differential cardiovascular responses to a CS as a function of the dorsal-ventral extent of mPFC lesions, suggesting that the functional contribution of mPFC may differ along this axis rather than being exclusively confined to PL vs. IL (Frysztak and Neafsey, 1991, 1994).

On the basis that animals with mPFC damage display extinction impairments (Morgan et al., 1993), a subsequent study sought to directly compare the effects of damage restricted to different mPFC subregions and better define their contribution to extinction learning. It was found that while vmPFC lesions (encompassing IL and to some extent PL) do not impair extinction learning *per se*, they disrupted extinction recall. Importantly, this effect was not observed in sham operated animals or animals with lesions that spared the majority of IL. The authors suggest that IL neural activity in particular is involved in the consolidation of extinction learning (Quirk et al., 2000).

Many of these studies have formed the basis for the proposed dichotomy of function in the mPFC in which PL regulates fear expression and IL fear suppression. However, these findings are largely discrepant in nature with reports indicating increases, decreases, or no changes in learning following mPFC damage. Moreover, of particular interest, Holson (1986) and Morgan and LeDoux (1995) demonstrate that dorsal PL lesions produce a generalized *increase* in fear expression, indicating that an intact dorsal PL may actually function to *suppress* fear, which is at odds with the current view. In addition, while Quirk et al. (2000) suggest that IL neural activity is importantly involved in the consolidation of extinction memories, similar experiments have not replicated these effects insofar as mPFC lesions do not yield deficits in either conditioned inhibition or extinction learning under some conditions (Gewirtz et al., 1997; Garcia et al., 2006). Thus, it appears the mPFC is not necessary for the formation or retrieval of extinction memories under some circumstances and this may be partially influenced by factors such as the strain of the animals used in these experiments (Chang and Maren, 2010).

As noted above, it has been shown that both behavioral and autonomic responses to a CS are differentially modulated as a function of the dorsal-ventral extent of mPFC damage (Frysztak and Neafsey, 1991, 1994). These findings leave open the possibility that cell populations with overlapping function exist in PL and IL. A more general interpretation of these lesion studies may be that the observed functional differences are a product of the lesion technique and size. It is possible that the behavioral effects reflect a shift in function along the dorsal-ventral axis, although this may not be solely interpreted as a functional opposition between PL and IL. It is worth noting that PL shows changes in laminar organization and cytoarchitectonic features along this axis which transitions into IL (Heidbreder and Groenewegen, 2003; Perez-Cruz et al., 2007; van de Werd et al., 2010). Hippocampal input to the mPFC is not uniform along this axis (Jay et al., 1989; Jay and Witter, 1991) and these differences may influence the behavioral outcome of localized damage. Overall, despite the controversies around the conclusions one can draw from these lesion studies, they have been instrumental to our understanding of the fear circuit and have led to a rapid increase in additional studies examining the mPFC in fear.

### Pharmacological and Microstimulation Studies

In an attempt to further characterize the role of PL and IL in fear, many studies have used pharmacological agents to temporarily inactivate the mPFC during behavioral tasks. These methods allow for circuit manipulation at discrete time points. For example, intra-PL infusion of the Na+ channel blocker tetrodotoxin prior to fear conditioning does not disrupt the acquisition of conditional fear, but reduces fear expression to a CS or context previously paired with shock (Corcoran and Quirk, 2007). Consistent with PL activity being necessary for fear expression, inactivation of PL, with the GABA-A receptor agonist muscimol, prior to extinction training also impairs fear expression (Laurent and Westbrook, 2009; Sierra-Mercado et al., 2011). However, this manipulation has no long-term effect on extinction recall, suggesting PL inactivation does not interfere with the acquisition of extinction (Laurent and Westbrook, 2009; Sierra-Mercado et al., 2011). Collectively, these findings suggest that PL activity underlies fear expression, but not learning *per se*.

There is some evidence to support the idea that PL signaling plays a role in aversive learning, beyond its role in fear expression, however, and this may extend to more dorsal regions, including ACC. For example, PL microstimulation increases fear expression while preventing successful extinction (Vidal-Gonzalez et al., 2006), implying that PL signaling shunts extinction learning by elevating fear. In addition, transient inactivation of rostral ACC impairs fear learning whereas activation enhanced fear acquisition and expression (Bissière et al., 2008). Interestingly, in a study in which rats were trained in a contextual bi-conditional discrimination task (in context A, one CS is paired with shock while a second CS is not, and this contingency is reversed in a second context) PL inactivation interfered with both the encoding and expression of appropriate CS responding. This suggests that PL may integrate contextual information to inform both learning and responding to conditioned stimuli (Sharpe and Killcross, 2014). Moreover, PL inactivation disrupts both recent and remote contextual fear memories after brief memory retrieval, indicating that PL signaling may be involved in reconsolidation. This reconsolidation blockade also prevented reinstatement, further showing that PL activity may subserve the reactivation of fear memories and contribute to their long-term maintenance (Stern et al., 2014), expanding the role of PL in the fear circuit. In summary, PL signaling appears to be a key component encoding the acquisition and expression of learned fears and this may vary based on specific task parameters.

While the PL appears to be involved in the expression of fear, it is widely believed that IL is involved in the suppression of fear during extinction learning and retrieval. IL inactivation increases freezing to conditioned tones while impairing within-session extinction and retrieval in both rats and mice (Sierra-Mercado et al., 2006, 2011; Laurent and Westbrook, 2009; Morawska and Fendt, 2012; Sangha et al., 2014). Additionally, conditioned tones paired with IL electrical stimulation enable low levels of freezing in rats that had not been previously extinguished, suggesting that IL activation is sufficient to mimic extinction training (Milad and Quirk, 2002; Milad et al., 2004). Interestingly, IL stimulation paired with presentation of a CS in anesthetized rats mimics the behavioral experience of extinction training (Park and Choi, 2010). These effects are frequency-dependent: high-frequency IL stimulation immediately after fear memory retrieval reduces freezing at a later time point, whereas low-frequency stimulation impairs extinction learning (Maroun et al., 2012; Shehadi and Maroun, 2013). This may reflect IL potentiation vs. depression with high- and low-frequency stimulation, respectively. In line with these studies, IL activation, via infusion of the GABA-A receptor antagonist picrotoxin, rescues extinction learning in extinction-deficient mice (Fitzgerald et al., 2014). Others have shown that IL activation prior to an extinction session dampens the expression of fear (Chang and Maren, 2011) and subsequently enhances extinction recall (Thompson et al., 2010; Chang and Maren, 2011).

Extinction learning produces a labile suppression of fear that is susceptible to relapse when a previously extinguished cue is presented outside the extinction context (i.e., fear renewal; Bouton, 2000, 2002; Bouton et al., 2006; Hermans et al., 2006; Maren et al., 2013; Vervliet et al., 2013; Goode and Maren, 2014). This process is likely mediated by hippocampal-prefrontal circuits (Corcoran and Maren, 2001; Maren et al., 2013). In addition, the timing of extinction trials relative to conditioning is also a key factor governing the long-term success of extinction training (Maren and Chang, 2006; Myers et al., 2006; Maren, 2014). Extinction trials delivered soon after conditioning often result in a failure to retain this memory long-term, which may reflect impaired mPFC signaling. Using an immediate extinction paradigm, intra-IL picrotoxin abolished conditioned freezing during extinction training and promoted a faster reduction of conditioned responding the following day (Chang and Maren, 2011). In a separate study, IL electrical stimulation paired with CS presentations limited the spontaneous recovery of fear the following day, rescuing the immediate extinction deficit (Kim et al., 2010). Collectively, these findings support the idea that IL signaling promotes extinction learning and suppresses conditional fear.

Overall, the findings discussed above generally lend support to a division of labor in which PL and IL are functionally opposed. However, due to the physical proximity of PL and IL, it is difficult to restrict infusions or electrical stimulation to only one subdivision. Moreover, pharmacological manipulations lack cell specificity, affecting both principal cells and interneurons in a similar fashion. Additionally, electrical stimulation results in ortho- and antidromic signaling which clouds the interpretation of directionality and localization of these effects. Given these experimental limitations, it is not surprising that there is evidence that challenges the dichotomous role of PL and IL in fear expression and suppression, respectively. For example, if PL activity underlies fear expression to associative stimuli, then PL activation at any time point of associative fear learning should increase freezing behavior whereas inactivation should impair freezing. Curiously, PL inactivation does not affect freezing under some conditions (Bravo-Rivera et al., 2014; Sharpe and Killcross, 2015) suggesting that ongoing freezing behavior is not solely dependent on PL activity and that other neural structures can compensate in its absence. Similarly, if IL is a necessary component of fear suppression, then IL activation should serve to promote extinction learning and subsequently reduce fear responding while inactivation should have the opposite effect. Interestingly, some studies have reported *facilitated* extinction learning with IL inactivation in both aversive and appetitive conditions (Akirav et al., 2006; Mendoza et al., 2015) making it possible that cell populations within IL exist that can bi-directionally modulate extinction learning. These findings challenge existing models of PL and IL function in fear and leave open the possibility that there is some functional overlap between PL and IL that allows one structure to compensate for the other under some conditions.

## mPFC Neural Correlates of Fear and Extinction

### Immediate Early Genes

Immediate early genes (IEGs) such as c-fos, Arc and Zif268 are activated in response to cellular stimulation, providing an indirect measure of neural activation and have been implicated in learning and memory (Davis et al., 2003; Plath et al., 2006). Interestingly, patterns of mPFC gene expression may be context-dependent, possibly as a result of feed-forward information being integrated from the hippocampus. In line with the idea that mPFC IEG expression may be partly modulated by context, PL and IL exhibited opposing patterns of Fos expression in a renewal paradigm in which an extinguished CS is presented in the extinction context (low fear) and in a different context (high fear). PL showed robust increases in Fos expression during fear renewal whereas presentation of the extinguished CS in the extinction context induced increased Fos expression in IL (Knapska and Maren, 2009). Similarly, in a separate set of studies, levels of Zif268 were greater in PL upon contextual fear recall (Stern et al., 2014), whereas increased IL Zif268 expression has been reported in animals recalling a remote cued fear memory; this effect was not observed in PL (Fitzgerald et al., 2015b). In addition, prefrontal levels of Arc mRNA expression show context specificity, with higher levels in BA, LA and IL of extinguished rats (Orsini et al., 2013). Further supporting a role for IL in extinction learning, extinction-deficient mice display reduced Fos and Zif268 expression in IL, implying that reduced IL activity may underlie this behavioral deficit (Hefner et al., 2008). In summary, these data suggest that PL and IL IEG expression displays context specificity with PL being primarily activated in a high fear state whereas IL is activated in a low fear state. These findings indicate that the mPFC may integrate contextual cues to process the meaning of the CS and inform conditioned responding.

The above IEG studies mainly suggest opposing roles for PL and IL in the expression or suppression of fear, respectively, while having little influence on learning *per se*. However, it has been shown that both PL and IL exhibit increased levels of Fos after conditioning, implying that PL and IL activity may underlie new learning. Interestingly, conditioning induced greater activation of PL and IL compared to extinction learning, and Fos expression following each session was indistinguishable between brain regions (Morrow et al., 1999; Herry and Mons, 2004). This conditioning-induced increase in Fos expression may partly be a response to the unconditioned footshock, rather than associative learning *per se*. However, an antisense oligonucleotide against c-fos mRNA, injected simultaneously into both PL and IL 12 h prior to conditioning, attenuated fear responses during an extinction session (Morrow et al., 1999). Thus, PL and IL appear to be involved in the acquisition of conditioned fear and to a lesser extent, are activated following extinction learning. It is worth noting that this effect was seen with simultaneous manipulations to PL and IL (Morrow et al., 1999), implying that there is some level of functional overlap between the two regions. However, the authors did not manipulate PL or IL alone, leaving the possibility that the decreased fear responding during extinction may be preferentially driven by one of these two regions. In support of the idea that PL and IL may covary at times, a separate study has shown that Fos and Zif268 expression were similar after the retrieval of both a recent and remote contextual fear memory (Frankland et al., 2004). These studies suggest that PL and IL can fluctuate similarly during the acquisition, extinction and expression of conditional fear.

As mentioned previously, animals subjected to extinction trials soon after conditioning often spontaneously recover high levels of freezing the following day which may result from impaired mPFC function (Maren and Chang, 2006; Maren, 2014). In support of this hypothesis, rats extinguished 15 min after conditioning displayed a general decrease in Fos expression in both PL and IL when compared to animals extinguished 24 h after conditioning (Kim et al., 2010; but see Stafford et al., 2013). This suggests that some basal level of activity in both regions is necessary for extinction learning. Additionally, others have shown that the spontaneous recovery of fear after extinction is associated with reduced Fos and Zif268 induction in both PL and IL of rats (Herry and Mons, 2004). Collectively, these studies further demonstrate that neuronal activity in PL and IL are positively correlated under some conditions. The observed similarities may stem from similar synaptic inputs and cortico-cortical interactions, although this remains an open question.

### Electrophysiology

#### Single-Unit Recordings

Electrophysiological methods also provide insight into the function of PL and IL neurons during the conditioning and extinction of fear. Using *in vivo* single-unit recordings in awake, behaving rats, Milad and Quirk (2002) provided the first evidence that CS-evoked responses in IL correlate with successful extinction recall. This study showed that IL neurons preferentially responded to a CS when rats successfully retrieve an extinction memory, but not during conditioning or the initial extinction session. This effect was specific to IL, as it was not seen in neurons recorded in PL or the medial orbital cortex. The authors suggested that extinction consolidation may enhance IL activity and this subsequently reduces fear the following day (Milad and Quirk, 2002). In agreement with this, successful extinction correlates with high-frequency IL bursting (Burgos-Robles et al., 2007), and under conditions in which extinction fails (i.e., immediate extinction) IL bursting is diminished (Chang et al., 2010). These *in vivo* findings have been complemented by *in vitro* studies, which have also provided support that IL signaling is altered upon extinction learning. For example, in slice preparations, the intrinsic excitability of IL neurons was decreased for up to 4 h after conditioning and this can be reversed with extinction training (Santini et al., 2008; Cruz et al., 2014). This reversal suggests the acquisition of extinction induces a ramping upward of spike firing during the consolidation phase, although this inhibition returned in rats that spontaneously recovered fear (Cruz et al., 2014).

How extinction learning and recall are precisely computed at the circuit level is not fully understood, although this was previously thought to be mediated by a direct IL→ITC pathway (Royer et al., 1999; Royer and Paré, 2002; Pape and Pare, 2010; Duvarci and Pare, 2014). In support of this idea, the ITCs are strongly responsive to IL stimulation in anesthetized rats (Amir et al., 2011). Interestingly, at basal levels of activity, ITC neurons actively inhibit each other; however, with brief IL stimulation the ITCs display increased firing rates which diminishes CeA output, a potential mechanism for reduced fear output (Li et al., 2011). Recent evidence, however, has suggested that IL exhibits low levels of connectivity to the ITCs (Gutman et al., 2012; Pinard et al., 2012; Strobel et al., 2015) bringing question to this proposed mechanism of extinction learning. These findings have prompted an updated hypothesis that posits disynaptic projections from IL to the ITCs via the BLA serve to engage inhibitory processes involved in extinction (Strobel et al., 2015). These disynaptic projections may be necessary for IL to overcome the inter-ITC inhibitory network in order to promote extinction learning and reduce fear. Overall, these data support a role for IL excitability in successful extinction learning.

Given that the PL has been implicated in the acquisition and expression of conditioned fear, it follows that this should be reflected in single-unit activity in awake, behaving animals. It has been reported that sustained spike firing in the PL during aversive CSs correlates with ongoing freezing behavior (Burgos-Robles et al., 2009). Consistent with this, extinction-deficient 129/S1 mice show elevated CS-evoked responses in PL, although this effect was also mirrored in IL (Fitzgerald et al., 2014). In contrast, others have reported that the expression of freezing behavior is associated with robust CS-evoked responses in IL (Chang et al., 2010; Fitzgerald et al., 2015b). Interestingly, Chang et al. (2010) also found that, in contrast to IL, CS-evoked PL activity was attenuated during fear expression, revealing a reciprocal relationship between PL and IL activity in the opposite direction to that predicted by prevailing models. In a recent study, we examined the pattern of spontaneous firing in simultaneously recorded PL and IL neurons immediately after fear conditioning (Fitzgerald et al., 2015a). In this post-conditioning period, rats exhibit sustained and high levels of fear that persisted for the duration of the 1 h recording session. During this transition from a low-fear to a high-fear state, spontaneous firing rates some neurons in PL and IL were transiently excited in the minutes following conditioning, but returned to basal levels soon after, despite ongoing freezing behavior. Interestingly, spontaneous firing rates of other neurons in IL were persistently suppressed over the duration of the post-conditioning period (Fitzgerald et al., 2015a). Collectively, these data suggest that PL spike firing alone is unlikely to mediate sustained freezing behavior; indeed, the expression of fear may be due, at least in part, to suppression of IL activity (Chang et al., 2010; Fitzgerald et al., 2015a).

Interestingly, similar to IEG studies, there is evidence for positively correlated single-unit activity in PL and IL after the conditioning or extinction of fear. For example, during the expression of conditioned fear (high fear), spontaneous firing rates are suppressed in both IL and PL, although IL suppression was more robust (Fitzgerald et al., 2015a). Additionally, Holmes et al. (2012) reported no differences in PL vs. IL CS-evoked responses throughout extinction learning as well as extinction retrieval. In a separate study, comparable conditioning-induced increases in CS-evoked activity were observed in the PL and IL of extinction-deficient 129/S1 mice (Fitzgerald et al., 2014). This provides further evidence that PL and IL may covary in their response properties at the single-neuron level, at least under some conditions. Other experiments have found that PL and IL neurons exhibit similar firing patterns in response to CSs or contexts associated with shock (Baeg et al., 2001) or in relation to the types of behavioral responses animals emit (e.g., freeze or move) in response to aversive CSs (Halladay and Blair, 2015). Hence, single-unit activity in IL and PL fluctuates similarly under a number of conditions, which is not surprising given their similar afferent inputs.

#### Local Field Potentials

In addition to single-unit recordings, LFP recordings suggest a high degree of synchrony between the mPFC, amygdala, and hippocampus throughout different stages of aversive learning. LFPs are generated by finely tuned synaptic input patterns, and recent studies have focused on LFPs at the circuit level as a mechanism by which distant brain regions effectively communicate. The coupling and synchronization of brain regions within the fear circuit are likely involved in memory formation and retrieval. Importantly, theta oscillations act to coordinate regional synchronization, providing a means of timely and efficient transmission of information. For example, the BLA and mPFC show enhanced theta synchrony during sleep after conditioning, which plays a role in memory consolidation (Popa et al., 2010). In line with this, increased BLA-mPFC theta synchrony has been observed in animals that successfully learned to differentiate between safe and aversive conditions (Likhtik et al., 2014). During learned safety, BLA firing activity was entrained to theta input from mPFC, suggesting that the BLA is selectively tuned to mPFC input, a potential mechanism underlying memory recall and thus behavioral responding (Likhtik et al., 2014). mPFC projections excite BLA neurons, indicating that inhibition of CeM output may be mediated by an active gating mechanism downstream of BLA (Likhtik et al., 2005). The directionality of this effect supports the role of mPFC in regulating amygdala activity, although it is well known that amygdala output influences mPFC function as well (Senn et al., 2014) and inactivation of BLA decreases PL activity (Sotres-Bayon et al., 2012). One study has shown that in male mice, PL and IL display opposing patterns of theta power across extinction, which may reflect new learning. Given their physical proximity and similar input it is somewhat surprising that LFPs would be drastically different between the two regions. Interestingly, this effect was not seen in females as they displayed heightened freezing and persistently increased mPFC theta in both PL and IL (Fenton et al., 2014). In addition, PL gamma power is elevated in extinction-deficient mice compared to mice that successfully extinguished (Fitzgerald et al., 2014). Moreover, other work has reported theta synchrony of an expanded network involving CA1-LA-IL during the retrieval of conditioned fear. Theta synchronization declined with extinction training, but was partially restored upon extinction recall (Lesting et al., 2011). In summary, LFPs may importantly affect the fear circuit at a global level and theta interactions might provide a mechanism for the fine-tuned organization of neural pathways underlying memory formation and recall.

## Optogenetics and Chemogenetics: Causal Mechanisms of Fear

The acquisition and retrieval of memories depend on complex patterns of neural activity from distinct neuronal populations defined by their genetic markers. Whereas much of the above evidence convincingly demonstrates a role of mPFC in fear, electrophysiology is only correlative and inactivation methods lack cellular specificity. As such, the fear-related causal mechanisms of precise neural activity and the contribution of different cell types remain largely unknown. Optogenetics and chemogenetics are virally-mediated techniques allowing for cell and circuit specific manipulations to selectively excite or suppress specific neuronal populations. Briefly, optogenetics requires the expression of exogenous light-sensitive ion channels to modulate neuronal activity with high temporal precision (Boyden et al., 2005; Fenno et al., 2011). One chemogenetic approach makes use of Designer Receptors Exclusively Activated by Designer Drugs (DREADDs), which are synthetic G-protein coupled receptors that respond selectively to the systemic injection of an inert ligand, clozapine *N*-oxide (CNO; Dong et al., 2010; Urban and Roth, 2015). These technologies provide an *in vivo* mechanism to control cellular physiology in intact neural circuits and delineate the causal contribution of specific neuronal subtypes to learning and memory.

Recently, optogenetic methods have been used to explore plasticity in prefrontal projections to the amygdala after fear conditioning. Combining optogenetics and *ex vivo* electrophysiology, Arruda-Carvalho and Clem (2014) have shown that in behaviorally naïve mice, the synaptic connectivity of IL and PL projections onto BLA principal neurons were similar. However, fear conditioning led to a decrease in inhibitory-excitatory balance in PL, but not IL. These data suggest that a PL→BLA pathway is crucial for encoding fear memories and may be engaged when encountering the CS at a later time point to promote a high fear state (Arruda-Carvalho and Clem, 2014).

As discussed above, extinction learning is thought to involve feed-forward inhibition that blunts CeA output via the ITCs (Royer et al., 1999), with IL synaptic transmission regulating this pathway via the BLA. The direct role of mPFC, however, had not previously been tested, including differences between PL and IL. One possibility is that, while weak in number, direct IL projections to the ITCs increase in strength with extinction training to inhibit the CeA, or IL projections to the BLA are modulated which ultimately influences ITC output. If so, the synaptic strength of this pathway may be causally linked to both the acquisition and recall of extinction. Using *ex vivo* electrophysiology and the excitatory optogenetic virus channelrhodopsin restricted to principal cells under control of the CAMKII promoter, Cho et al. (2013) demonstrated that extinction learning *reduced* synaptic efficacy in BLA projecting mPFC neurons. Interestingly, mPFC synaptic transmission to ITCs was *unchanged* and thus the overall balance in the mPFC-BLA pathway shifted towards inhibition following extinction. This effect may stem from monosynaptic connections to BLA interneurons. The authors note that PL and IL projections were nearly indistinguishable in terms of location and evoked current amplitudes downstream in BLA, with the most robust projections terminating in the anterior subdivision of BLA, and to a lesser extent on the ITCs. It could be that the weak IL→ITC projections can dampen amygdala output, without a measurable change in synaptic strength. The relative shift in balance towards BLA inhibition may in turn promote ITC activity, thus impeding CeA output and dampening fear (Cho et al., 2013). These findings suggest a high degree of similarity between both the structural and functional components of PL and IL, lending support to the hypothesis that these regions may covary as noted in several other reports (Baeg et al., 2001; Herry and Mons, 2004; Kim et al., 2010; Holmes et al., 2012; Halladay and Blair, 2015).

In a similar fashion, Hübner et al. (2014) explored functional connectivity between mPFC and the amygdala using retro-bead tracing and excitatory optogenetic techniques in behaviorally naïve mice. They further confirm that mPFC sends monosynaptic excitatory projections to both principal cells and interneurons in the basomedial nucleus of the amygdala (BM). Activating these inputs resulted in feed-forward inhibition of both principal cells and more frequently interneurons, promoting a disinhibition of BM principal cells. PL and IL similarly excited principal BM neurons, consistent with previous work (Cho et al., 2013) and received comparable feed-forward inhibition from amygdala feedback loops. However, this study suggested that IL inputs target mainly non-fast spiking interneurons (Hübner et al., 2014). This discrepancy may be explained by the fact that these findings were in behaviorally naïve mice as compared to mice undergoing extinction training in Cho et al. (2013). As noted, the basal levels of synaptic strength in mPFC-BLA circuits may shift significantly after behaviorally relevant events making it difficult to interpret these current findings in regard to fear. Nonetheless, these data further contribute to a growing body of evidence surrounding structural and functional similarities between PL and IL.

Optogenetic manipulations of specific monosynaptic pathways have provided evidence for a revised hypothesis of IL-mediated signaling in extinction. As mentioned above, it was previously believed that the ITCs were a major target of IL projections. A more recent model has proposed that this pathway is disynaptic with BLA serving as the interface between IL and the ITCs (Strobel et al., 2015) given that the direct IL-ITC connections are weak and not modulated upon extinction training (Gutman et al., 2012; Pinard et al., 2012; Cho et al., 2013). It has previously been demonstrated that pharmacological activation of the IL during extinction enhances long-term retention (Thompson et al., 2010; Chang and Maren, 2011) and that CS-evoked activity correlates with extinction recall (Milad and Quirk, 2002). While it was assumed that these findings were a product of enhanced synaptic transmission of pyramidal cells, this had not been tested directly *in vivo*. In a recent study it was shown that optogenetically activating IL projection neurons during extinction reduces fear expression and enhances extinction recall the next day, in the absence of optical stimulation (Do-Monte et al., 2015). Silencing the same neuronal population during extinction had no within-session effect, but impaired retrieval the following day, consistent with the idea that IL activation during extinction learning predicts the extent of retrieval. Curiously, optogenetically inhibiting IL during extinction retrieval had no behavioral effect (Do-Monte et al., 2015), in contrast with what the findings of Milad and Quirk (2002) would predict.

A similar study, examining the pathway specificity of this effect has found evidence in support of the idea that IL signaling is important for the formation, but not the recall of extinction memories (Bukalo et al., 2015). In this study, the authors selectively expressed either the excitatory opsin (ChR2) or inhibitory opsin (ArchT) in glutamatergic vmPFC neurons (restricted primarily to IL). Optogenetic activation of vmPFC-amygdala projecting neurons during a “partial” extinction session (10 CS alone trials) was sufficient to promote long-term facilitation of extinction learning, yielding low levels of freezing the following day in the absence of optogenetic stimulation. In contrast, inhibiting this pathway during extinction training yielded long-term deficits in extinction memory formation, providing evidence that activation of the vmPFC→BLA pathway is a necessary component underlying extinction. Interestingly, optogenetic activation or inhibition of this pathway during extinction retrieval did not alter freezing behavior relative to controls, suggesting that vmPFC afferents in the amygdala do not regulate memory retrieval (Bukalo et al., 2015). It is worth noting that in both of these studies (Bukalo et al., 2015; Do-Monte et al., 2015), the retrieval tests were conducted with very few (4–5) test trials. This test procedure would be expected to yield substantial spontaneous recovery and limit IL engagement. It is possible that inhibiting IL or its BLA afferents over a longer (multi-trial) test session would reveal an effect of vmPFC inactivation on extinction retrieval.

A key question of interest that can be addressed with viral technologies lies with the ability to selectively target and modulate neuronal subtypes based on protein expression. Parsing the role of genetically defined interneurons can inform us about local modulatory mechanisms and how this impacts the extended fear network. For example, optogenetic inhibition of dmPFC (encompassing ACC/PL) PVINs causally initiated freezing behavior in unconditioned animals and also modulated fear expression in previously conditioned animals (Courtin et al., 2014). These interneurons can be further subdivided into fast-spiking and non-fast spiking interneurons based on firing rate properties. Fast-spiking PVINs target the perisomatic region of pyramidal cells, thereby dictating the timing and synchronization of action potentials (Cobb et al., 1995; Freund and Katona, 2007). Thus, inhibiting dmPFC PVINs can disinhibit and synchronize the firing of projection neurons. This synchronization is crucial to regulating timely and efficient transmission of information to drive the appropriate behavioral response. These data indicate a key role for PVINs in determining freezing behavior by disinhibiting dmPFC (Courtin et al., 2014). It is unknown, however, if this mechanism is specific to dmPFC regulating conditioning and fear recall. For instance, would activating these neurons induce renewal in an extinction context? A second question to address lies at the circuit level: what influences the state of dmPFC PVINs? Gabbott et al. (2006) have demonstrated that BLA output monosynaptically innervates mPFC PVINs—could this effect be driven by feed-forward disinhibition from amygdala projections? Additionally, ventral hippocampal projections also alter firing patterns of putative mPFC interneurons (Sotres-Bayon et al., 2012), so perhaps amygdala and ventral hippocampal projections to mPFC act to synchronously disinhibit PL output. Alternatively, is direct optical activation of PL pyramidal cells sufficient to induce freezing behavior and is this local modulatory mechanism conserved between brain regions? For example, would disinhibiting IL pyramidal cells induce locomotor behavior? While currently unknown, optogenetics provide the ability to answer such questions by controlling neural activity in a cell and circuit specific manner.

Chemogenetic technology is also beginning to contribute to our understanding of mPFC physiology. By expressing an excitatory DREADD virus in dmPFC (encompassing ACC/PL), Yau and McNally (2015) have recently shown that increased activation of this region is causally involved in prediction error. In fear conditioning, animals must use information from the past to predict the meaning of a CS. If the animal expects the US to be delivered and it is not, this produces a large prediction error. Using a blocking design in which animals are trained to fear one CS and then later given compound training (CS1 and a novel CS2), learning about CS2 will be blocked under normal conditions. However, dmPFC activation with a virus infecting all cell types or a virus restricted to pyramidal neurons was sufficient to promote learned fear to the second CS. Thus, dmPFC activation promotes the acquisition of conditioned fear under circumstances where learning would not otherwise occur. Importantly, this was not simply due to increased fear expression independent of learning (Yau and McNally, 2015). Given the results discussed above, it is somewhat surprising that this manipulation alone did not induce freezing behavior. If disinhibiting dmPFC optogenetically was sufficient to increase freezing, then directly activating it should have an even greater effect. This may be due to differences in the level of viral expression at the time of testing or to differences in activating neuronal activity directly through ion channels vs. G-protein coupled receptors. In summary, optogenetic and chemogenetic technologies have only begun to add to our understanding of the role of mPFC in the fear circuitry, and are primed to contribute further.

## Neuroimaging and Human Homologs

The neural circuits underlying fear conditioning and extinction in rats have also been identified in humans. For example, the dorsal anterior cingulate cortex (dACC) and the ventromedial prefrontal cortex (vmPFC) have been proposed to regulate the expression and suppression of fear in humans, respectively. While the temporal and spatial resolution of neuroimaging techniques cannot provide fine anatomical details for cross species comparison, they have provided a broad look at the human fear circuit and insight into PTSD. Using functional imaging with a standard fear conditioning paradigm, Phelps et al. (2004) reported activation of the vmPFC that corresponded with the expression of fear during extinction learning. Interestingly, individuals with PTSD often display decreased mPFC blood flow upon recalling a traumatic experience which likely disrupts extinction learning (Semple et al., 1996; Bremner et al., 1999; Shin et al., 1999). In humans, vmPFC has an inhibitory influence over the amygdala similar to that in rodents (Delgado et al., 2008). The vmPFC-amygdala pathway may be dysregulated in some cases of PTSD (Gilboa et al., 2004; Garfinkel et al., 2014) and patients with bilateral vmPFC damage present heightened amygdala activation to aversive images (Motzkin et al., 2015). Thus, vmPFC regulation of amygdalar output may be a common circuit underlying fear extinction.

Another possibility is that those who suffer from PTSD fail to use contextual cues to appropriately guide behavioral responding, resulting in a greater degree of generalized fear (Maren et al., 2013; Garfinkel et al., 2014). This is more likely mediated by vmPFC-hippocampal networks and indeed, individuals with PTSD often have decreased hippocampal volume (van Rooij et al., 2015). Studies in healthy volunteers show that vmPFC-hippocampal activation correlates with extinction success and that this activation is context dependent (Kalisch et al., 2006; Milad et al., 2007b). This network displays diminished activity in PTSD patients, further contributing to extinction deficits (Milad et al., 2009). Structural studies have shown that cortical thickness of vmPFC correlates with the degree of extinction retention in healthy individuals (Milad et al., 2005), providing evidence that neural mechanisms of extinction may be conserved across species, although this has not been replicated in a related study (Hartley et al., 2011). It is unclear if these potential structural differences precede the development of PTSD or if they are a consequence of the traumatic experience. A recent study suggests the former in that combat-exposed veterans who did not develop PTSD showed no differences in hippocampal volume compared to healthy controls (van Rooij et al., 2015). In summary, dysregulated vmPFC activity may be a common biomarker of fear and disrupted extinction learning across species.

The dACC has received considerable attention for regulating fear expression. In healthy subjects, cortical thickness of dACC is positively correlated with skin conductance responses during fear conditioning and this brain region is activated by a CS (Milad et al., 2007a). Interestingly, in a separate study, during extinction training, amygdala metabolism *positively* predicted vmPFC activation while *negatively* predicting dACC activation, and resting dACC metabolism predicted fear expression (Linnman et al., 2012a,b). dACC-amygdala networks have also been reported during fear memory consolidation (Feng et al., 2013, 2014) and dACC shows sustained activity increases when shock delivery was expected (Linnman et al., 2012b). Thus, dACC signaling may correspond to ongoing fear responses and it has been shown that PTSD patients display a greater activation of dACC during extinction recall (Milad et al., 2009). This hyperactivity was larger in men with PTSD, implicating the mPFC in sex differences underlying the disorder (Shvil et al., 2014). Overall, there is a growing body of evidence supporting distinct roles within the mPFC regulating emotional learning and memory in humans. However, many of these brain imaging studies do not directly report data comparing vmPFC and dACC, leaving the possibility of covariation of these two brain regions virtually unexplored at the level of human fear conditioning and PTSD.

## Parallels with Reward and Drug Seeking Behavior

Given the recent challenges to the precise role of the mPFC in fear, it is worth turning to the appetitive literature to draw parallels and perhaps provide a more integrated view on mPFC function. In both food- and drug-motivated instrumental tasks, the PL and IL have been posited to play different roles in conditional responding (Peters et al., 2009). Specifically, the PL has been posited to drive drug seeking behavior (McFarland and Kalivas, 2001; Capriles et al., 2002), whereas the IL may suppress conditional responding after extinction (Peters et al., 2008; Moorman et al., 2014). In other words, the PL is believed to be required for the execution of goal-directed behavior (“go”), whereas the IL is believed to regulate behavioral inhibition (“stop”). This view of medial prefrontal cortical function in appetitive instrumental conditioning paradigms has considerable homology with the canonical view of mPFC function in the fear conditioning and extinction (Peters et al., 2009).

In addition to regulating goal seeking and response inhibition, the PL and IL appear to regulate different forms of instrumental responding over the course of conditioning. During instrumental conditioning, performance early in training typically reflects goal-directed behavior (i.e., actions), but this shifts to outcome-independent (e.g., habitual) performance after extended training. Interestingly, rats with PL lesions exhibit habitual responding that is insensitive to outcome value both early and late in training, whereas rats with IL lesions exhibit goal-directed responding even after extended training (Killcross and Coutureau, 2003). These data suggest that PL promotes flexible, goal-directed responding, whereas the IL inhibits flexibility and promotes behavioral rigidity and perseveration. In line with this idea, IL inactivation reinstates goal-directed responding in rats with extensive training and reduces habitual responding in a response-conflict task (Coutureau and Killcross, 2003; Haddon and Killcross, 2011).

However, recent evidence has surfaced that challenges the canonical view in which PL and IL serve opposing functions for reward/drug seeking behavior (Moorman et al., 2014). For example, there is emerging evidence that PL lesions or inactivation have no effect on reward seeking (Weissenborn et al., 1997; Capriles et al., 2002), and several investigators have shown that PL may serve an inhibitory role in reward/drug seeking under some conditions (Ishikawa et al., 2008; Jonkman et al., 2009; Hayton et al., 2010, 2011; Mihindou et al., 2013; Martín-García et al., 2014). For instance, cocaine self-administration *decreases* PL pyramidal cell excitability and optogentically activating PL pyramidal cells *reduces* drug seeking behavior, whereas optical inhibition of this same population of cells *increases* this behavior (Chen et al., 2013).

Similarly, conflicting results regarding the precise function of IL have also surfaced. IL inactivation has been shown to *decrease* the maintenance of responding as well as reinstatement of lever pressing for cocaine (Di Ciano et al., 2007; Pelloux et al., 2013; Vassoler et al., 2013). In addition, it has recently been shown that the vmPFC (encompassing IL) plays a role in the expression of cocaine seeking behavior (Koya et al., 2009), a role previously thought to rely primarily of PL signaling. The fact that IL can both activate (Koya et al., 2009) and inhibit (Peters et al., 2008) drug seeking behavior suggests a more complex role for the mPFC, which is not yet fully appreciated. In support of this, recent work has shown that the vmPFC plays a time-dependent role in both the expression and extinction of cocaine seeking (van den Oever et al., 2013). Moreover, a recent study that recorded single-unit activity in PL and IL found cue-evoked activity in both areas during reward seeking and extinction. The authors show that neurons in both areas encoded contextually appropriate behavior (initiation during reward seeking vs. withholding during extinction), suggesting that PL and IL integrate contextual information to regulate behavior, rather than opposing each other to encode go vs. no-go behaviors (Moorman and Aston-Jones, 2015). Despite similar response properties, it remains possible that PL and IL signaling may be coupled to different response outcomes regarding goal-directed vs. habitual behavior. This may partially explain the tendency of PL and IL neural activity to covary, but lesion and inactivation studies suggest some functional bias. Overall, these recent findings support the idea that cell populations within both PL and IL can serve to either activate or inhibit drug seeking behavior and suggest a more complicated interplay of PL and IL than previously thought.

One interesting point about the possibility of overlapping circuits for fear and addiction is the striking difference in behavior that has been suggested to be controlled by PL and IL. In fear, PL activation is thought to underlie fear expression, and in drug seeking PL is thought to encode the expression of drug seeking activity. The nature of these behaviors is quite different. That is, in a high fear state animals exhibit robust freezing (inhibition of movement) whereas the expression of drug seeking behavior corresponds to a rapid activation of movement. However, the associative structure and psychological processes underlying these behaviors may be similar. It has been shown that “sign-trackers” (rats who approach a food predictive cue) also show increased auditory fear (compared to context fear), suggesting that these animals are “cue-directed” (Morrow et al., 2015). These data suggest that overlapping circuits may be engaged independent of the behavioral outcome. In summary, emerging evidence suggests a more complex role for the mPFC in reward/drug seeking behavior, similar to that in fear, insofar as it remains possible that distinct subpopulations exist within both PL and IL that subserve similar function to either promote or inhibit behavior, which is likely biased by context. It seems unlikely that an entire region of PFC would be necessary for any given function; rather neuronal populations within the mPFC may ultimately underlie a particular behavior through similar afferent and efferent connections.

## Conclusions

Overall, the majority of the work summarized above has focused on a division of labor within mPFC, where its subregions work largely independently to bidirectionally regulate fear output. These mechanisms appear to be conserved across species. In particular, the canonical view has been that dorsal regions (PL/dACC) of mPFC regulate fear expression and ventral regions (IL/vmPFC) fear suppression. However, findings from recent studies challenge the underlying assumptions of this model. For example, a number of recent anatomical and electrophysiological studies have shown that PL and IL project similarly to the amygdala (Gutman et al., 2012; Pinard et al., 2012; Cho et al., 2013; Hübner et al., 2014) and that neuronal activity (IEG, LFPs, single-units) in IL and PL covary during the conditioning and extinction of fear (Morrow et al., 1999; Baeg et al., 2001; Frankland et al., 2004; Herry and Mons, 2004; Kim et al., 2010; Holmes et al., 2012; Fitzgerald et al., 2014, 2015a; Halladay and Blair, 2015). Moreover, there are conditions under which IL and PL activity show functionally dichotomous activity patterns during the expression or suppression of conditioned fear, but in a direction opposite to that predicted by the canonical model (Chang et al., 2010).

However, even when IL and PL activity covary, it remains possible that the downstream effect of this activity is functionally opposed due to the different efferent targets of each area. Moreover, PL and IL have known structural and functional interactions with each other (Hoover and Vertes, 2007; van Aerde et al., 2008; Ji and Neugebauer, 2012; Little and Carter, 2012, 2013) and these interactions may bias the output of either area despite similar engagement of both regions in a particular task. Another possibility that has been largely unexplored is that distinct neuronal populations within PL or IL may show functional redundancy, where some neurons within each area modulate fear output differentially (e.g., Halladay and Blair, 2015). Given the similar connectivity of PL and IL, this possibility cannot be excluded.

While a wealth of research has explored the role of the mPFC in fear, it is clear that the precise contributions and function of the IL and PL in fear conditioning and extinction are not yet fully understood. Additional experiments coupling electrophysiology with cell and circuit specific techniques are primed to further delineate the complex roles of PL and IL within the fear circuit. A more sophisticated approach looking at simultaneously recorded single-units and oscillatory processes in PL and IL may help to better parse the expanding role of the mPFC in fear. Furthermore, an advanced understanding of the functional input and output patterns of PL and IL can help disambiguate many of the discrepant results. It is likely that PL and IL serve to integrate contextual information to inform behavioral responding and that context greatly impacts the response properties of these two regions, as well as the complexity of the tasks, with more complex tasks requiring greater cortical input. Continued work will likely shed light on unresolved issues, providing translational value for the treatment of trauma-related disorders such as PTSD. An enhanced understanding of the fear circuit at the level of rodents and humans may provide novel insight to improve current therapeutic outcomes and dampen inappropriate fear responding.

## Author Contributions

TFG and SM wrote and edited the manuscript.

## Funding

Supported by a grant from the National Institutes of Health (R01MH065961) and a McKnight Memory and Cognitive Disorders Award to SM.

## Conflict of Interest Statement

The authors declare that the research was conducted in the absence of any commercial or financial relationships that could be construed as a potential conflict of interest.
